# Properties and Antioxidant Action of Actives Cassava Starch Films Incorporated with Green Tea and Palm Oil Extracts

**DOI:** 10.1371/journal.pone.0105199

**Published:** 2014-09-24

**Authors:** Kátya Karine Nery Carneiro Lins Perazzo, Anderson Carlos de Vasconcelos Conceição, Juliana Caribé Pires dos Santos, Denilson de Jesus Assis, Carolina Oliveira Souza, Janice Izabel Druzian

**Affiliations:** 1 Federal University of Bahia, College of Pharmacy, Department Food Science, Ondina, Salvador, BA, Brazil; 2 Federal University of Bahia, Department of Chemical Engineering, Federação, Salvador, BA, Brazil; RMIT University, Australia

## Abstract

There is an interest in the development of an antioxidant packaging fully biodegradable to increase the shelf life of food products. An active film from cassava starch bio-based, incorporated with aqueous green tea extract and oil palm colorant was developed packaging. The effects of additives on the film properties were determined by measuring mechanical, barrier and thermal properties using a response surface methodology design experiment. The bio-based films were used to pack butter (maintained for 45 days) under accelerated oxidation conditions. The antioxidant action of the active films was evaluated by analyzing the peroxide index, total carotenoids, and total polyphenol. The same analysis also evaluated unpacked butter, packed in films without additives and butter packed in LDPE films, as controls. The results suggested that incorporation of the antioxidants extracts tensile strength and water vapor barrier properties (15 times lower) compared to control without additives. A lower peroxide index (231.57%), which was significantly different from that of the control (p<0.05), was detected in products packed in film formulations containing average concentration of green tea extracts and high concentration of colorant. However, it was found that the high content of polyphenols in green tea extract can be acted as a pro-oxidant agent, which suggests that the use of high concentration should be avoided as additives for films. These results support the applicability of a green tea extract and oil palm carotenoics colorant in starch films totally biodegradable and the use of these materials in active packaging of the fatty products.

## Introduction

The interest in biodegradable films produced from natural sources has increased in recent years due to the concerns about the environment and the consumer demand for the improvement of overall product characteristics (quality and appearance) [1], [2]. The basic materials used to produce biodegradable films are polysaccharides, proteins and lipids compounds [3]. Regarding polysaccharides, starch can produce biodegradable films at low cost and on a large scale [4]. Furthermore, starch-based materials may contribute to utilization of nonrenewable resources and the environmental impact caused by synthetic plastics [4]. Global production of cassava has nearly doubled over the past 30 years to about 260 million tons in 2012, making it an abundant and attractive starch source for researchers. Over half is grown in Africa, with a third in Asia and 14% in Latin America. Nigeria and Brazil are the largest producers, growing about 50 and 25 million tons in 2012, respectively [5].

A number of recent studies have focused on extending the functional properties of biodegradable films by adding different natural compounds to yield a biodegradable totally bioactive packaging material [2], [3], [6]. Active packaging films with antioxidant properties, developed by incorporating active functional ingredients into packaging systems, can offer protection against chemical and biological contamination [7], [8], and can delay oxidative changes in packaged products containing fatty components [9].

Oxidation is one of the most common mechanisms of degradation in foodstuffs and can limit the shelf life of food [10]. This process can decreased nutritional quality, increased toxicity, development of off-odor, and altered texture and color. Consequently, the shelf life and sales this products decrease. The direct addition of antioxidants in products, especially in foods, in one large initial dose is limited by the potential for rapid depletion of the antioxidants, in addition to very high initial concentrations [11]. Producers and packaging companies can inhibit the food oxidation process by adding antioxidants compounds at pack. Several packing formulations using synthetic compounds, such as butylated hydroxytoluene and butylated hydroxyanisole, have been developed [12], [13]. However, modern consumer trends show increasing concern with the use of synthetic chemicals and the belief that natural antioxidants are safer and of greater nutritional benefit. Therefore, a need exists in the food industry to develop polymer packaging which can deliver natural antioxidants in a controlled manner throughout the product shelf life [11], [14], [15].

Research has focused on natural edible antioxidants, such as phenolic compounds, flavonoids [6], [12], [17], [18], [19] and carotenoids [8], [16], which are commonly found in natural sources, such as the green tea and palm oil and kernels.

Green tea is an excellent source of polyphenols, which are natural antioxidants that can be used as alternatives to synthetic antioxidants [17], [18], [19]. Polyphenols are trends to substitute them with naturally available antioxidants and can inhibit oxidation [20], [21]. Tea catechins can act as antioxidants by donating hydrogen atoms, by accepting free radicals, by interrupting chain oxidation reactions, or by chelating metals [22]. Wanasundara and Shahidi [23] suggested that the annexation of hydroxide groups to catechin molecules is the main factor that causes the strong antioxidant proprieties found in green tea extracts, reducing the formation of peroxides more effectively than BHT, BHA and a-tocopherol. It must be noted that some studies have suggested pro-oxidative proprieties of some polyphenols [24]. The activity of polyphenols depends on many factors, for example, the reductive potential, the chelating ability of the metals, the pH of the medium, solubility, bioavailability and stability in tissues and cells [21].

Carotenoids, such as α and β-carotene provide antioxidant protection because of their capacity to scavenge free radicals [25], [26], and palm oil reaching a world production of 50 million tons in 2012 [5] is a major sources [27]. The antioxidant activity of carotenoids in organic solutions is related to oxygen concentration, the chemical structure of the carotenoids, and the presence of other antioxidants [26], [27], [28].

The commercial use of edible films has been limited because these materials have poor mechanical and barrier properties as compared to synthetic polymers [29]. The successful use of natural compounds, such as phenolic and carotenoids, in packaging films is greatly dependent on the final characteristics of the films. The most important properties to be evaluated in biodegradable films are microbiological stability, adhesion, cohesion, wettability, solubility, transparency, mechanical properties, sensory and permeability to water vapor and gases. Once these properties are known, the composition and behavior of the material can be predicted and optimized [9].

Thus, studies to develop an innovative active food packaging that inhibits oxidation and behaves as a scavenger of oxygen radicals are of great interest [17]. The objective of this study was to develop films totally biodegradable from cassava starch containing green tea and palm oil carotenoids extracts, as actives natural compounds to be used as packaging fatty products, adding value to different agro-industrials chains.

## Experimental

### 2.1 Materials

Cassava starch (amylose –23.5% and amylopectin –64,2%) was donated by Cargill Agrícola S.A. (Porto Ferreira, SP, Brazil). Glycerol, analytical grade, was purchased from Synth S.A (Diadema, SP, Brazil). Green Tea *(Camellia sinensis)* was purchased from Mãe Terra Ltda (Osasco, SP, Brazil). Commercial colorant VEGEX NC 3c WSP mct extracted of the palm oil (*Elaeis guineensis*), containing 35% α-Carotene and 65% β-Carotene, was provided by Chr. Hansen (Hørsholm, Denmark). Commercial butter without antioxidant was obtained from Imperial (BA, Brazil). Low-density polyethylene (LDPE) film (0.020 mm thickness and 15.86×10^−8^ g_H2O_.mm/m^2^.h.kPa water vapor permeability) was purchased from local markets (Salvador, BA, Brazil).

### 2.2. Film Preparation

Preliminary experiments were conducted to evaluate the maximum concentrations of additives that could be incorporated to the films, in order to obtain homogeneous materials, flexible and easy to handle. Therefore, different concentrations of colorant (0.01, 0.05 and 1.00%) and green tea (2.5, 5.0 and 7.5%) were alternately tested. At the end of this stage, the maximum concentrations were fixed in 0.05% for colorant and 5.0% for the green tea. The other concentrations used did not show desirable characteristics in the films obtained.

For films production, film-forming dispersions was prepared by with an aqueous green tea extract obtained from green tea powder (0–5.0% of dry leaves, g/100 g) by method of percolation with 2 L hot deionized water (80°C) for optimal extraction and preservation of antioxidant compounds [30]. The extract was cooled to room temperature and then filtered through Whatman No.1 filter paper.

Then, the aqueous green tea extract was added into cassava starch (4.0%, g/100 g) of previously dried (40°C, 6 h), glycerol (1.0%, g/100 g) and colorant powder (0–0.05%, g/100 g) to form the starch-plasticizer dispersions with approximately 90 wt %(w/v) solid concentration. The colorant and green tea extract were added according to a 2^2^ central composite design, which was used to investigate the influence of two independent variables, namely, the concentrations of the colorants and the green tea extract. Film forming solutions were heated to 70°C, and the films were prepared by a casting technique, in which 66–67 g of the film-forming suspension was dehydrated on 150 mm diameter polycarbonate petri dishes kept at 30°C under renewable circulated air (Nova Etica, 400ND, SP, Brazil). All the dried starch film were preserved in a humidity chambers (25°C, RH = 75%) for further testing.

### 2.3. Film Characterization

The film was characterized by the thicknesses, total solid content, mechanical (tensile strength and elongation at break), barrier (water vapor permeability) and thermal properties (TGA).

#### 2.3.1. Film Thickness

The average film thicknesses of the preconditioned samples (75% RH, 25°C) were measured using a flat parallel surface external digital micrometer (Digimess, Ip40 0–25 mm, São Paulo, Brazil) with 0.001 mm resolution. Five replications were conducted for each sample treatment. Five measurements were taken at random positions around the film sample and the mean values were calculated.

#### 2.3.2. Mechanical Properties

Test filmstrips (8×2.5 cm) cut from preconditioned samples (25°C; 75% RH) were characterized for tensile strength resistance and elongation at break percentage by Universal Testing Instrument, electromechanical and microprocessor (EMIC, model DL-200MF, Instron, Paraná, Brazil). The tests were conducted according to the ASTM D882-00 method [31], [32]. Ten specimens were tested for each formulation.

#### 2.3.3. Water Vapor Permeability (WVP)

The samples were analyzed using the ASTM E96-80 method [31], modified by Gontard and others [33]. The relative humidity outside of the cell was fixed at 100% (pure water) and at 0% within the cell (dry silica). Four cells were prepared for each analysis and were weighed daily until a 4% weight gain of the silica was attained. Two control cells were prepared without the film and conditioned similarly. The weight gain of each cell was measured with time, and the water vapor permeability was calculated by equation 1 [34].
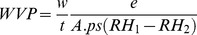
(1)


where *w/t* is calculated from the linear regression of the weight gain over time, *A* is the film area, *e* is the film thickness, *ps* is the vapor saturation pressure (kPa), *RH_1_* is the relative humidity inside the chamber and *RH_2_* is the relative humidity inside the cells.

#### 2.3.4. Thermogravimetric analyze (TGA)

To investigate the thermal stability of the films, curves were generated with a thermogravimetric analyzer (TGA) from Pyris 1 TGA (Perkin Elmer, Pyris, Shelton, USA). Samples of approximately 5–6 mg were tested in an atmosphere of nitrogen (20 mL min-1), and the temperature was increased at a rate of 20°C min^−1^ from room temperature to 600°C. The temperatures at which the rate of decomposition of the sample was at a maximum (Td) were obtained from thermogravimetric derivative curves (DTG).

### 2.4. Bio-Based Film used to Pack a Product

Butter was packed in cassava starch-plasticizer materials containing both actives green tea and carotenoids extracts colorant. Square-shaped films (5×2 cm–10 cm^2^) of 0.164 and 0.212 mm in thickness were molded (Sealer Sulpack SM 400 TE, Brazil) with the open top. Butter homogenized and congealed in blocks with 3×2 cm (10.00 g ±0.54) were involved in films, bubbles of oxygen were removed, and the film was sealed on top.

The antioxidant capacity and the stability of packaged butter during extended storage at 0, 7, 15, 30, and 45 days under storage conditions of accelerate oxidation (64% relative humidity at 30±2°C). The film storage with butter and analyses were carried out in a dark room to avoid the effects of light interference.

Unpackaged butter (C1), butter packaged in cassava starch-plasticizers without antioxidant additive (C2) and butter packaged in LDPE (C3) were used as controls.

### 2.5. Packaged Product Oxidative Stability

The oxidative stability from a packaged product was evaluated through the peroxide index, conjugated diene and total carotenoids of the butter, and these parameters were analyzed at 0, 7, 15, 30 and 45 days of storage.

#### 2.5.1. Peroxide Index (PI)

The peroxide index (PI) was determined by the titration method described by the Association of Official Analytical Chemists [35].

#### 2.5.2. Total Carotenoids Content (TC)

To measure the total carotenoids content (TC), 1.0 g of the packaged butter was dissolved in petroleum ether. The TC was determined spectrophotometrically at 440 nm (UV/Vis Spectrometer Lambda 20, Perkin-Elmer, Norwalk, Connecticut, USA) and was calculated according to equation 2 [36].
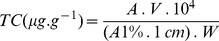
(2)


Where *TC* is the total carotenoids, *A* is the absorbance at 435 nm, *V* is dilution volume (mL), *A1%.1 cm* is the absorptivity coefficient value (2592) and *W* is the sample weight (g).

### 2.6. Actives Films Stability

The actives bio-based films stability used to pack butter was evaluated by analyzing the total carotenoids, total polyphenols and total flavonoids at 0, 7, 15, 30, 45 days of storage.

#### 2.6.1. Total Carotenoids Content (TC)

For total carotenoids (TC) values, films samples (1.00 g) were prepared according to Silva and Mercadante [37] and analyzed spectrophotometrically at 440 nm (UV/Vis Spectrometer Lambda 20, Perkin-Elmer, Norwalk, Connecticut, USA). The TC concentration was determined according to equation 2 at 440 nm.

#### 2.6.2. Total Phenolic Content (TP)

The total phenolic content (TP) of the film samples (100 mg) was extracted with water after centrifugation (4400 rpm/5°C/3 min; Eppendorf, 5702R, Hamburg, Germany), being evaluated at 0 and 45 days of storage. The TP in the supernatant was spectrophotometrically determined at 760 nm (UV/Vis Spectrometer Lambda 20, Perkin-Elmer, Norwalk, Connecticut, USA) using Folin-Ciocalteu reagent, and the results were expressed as gallic acid equivalents [8].

#### 2.6.3. Total Flavonoid Content (TF)

The total flavonoid concentration was measured using the same supernatant final sample of the total phenolic content. The final sample (1 mL) was added to a 10 mL volumetric flask containing 4 mL of distilled water. Then, 0.3 mL of 5% sodium nitrite solution was added to the volumetric flask, and 0.3 mL of 10% aluminum chloride was added after 5 min. One minute later, 2 mL of 1 M sodium hydroxide was also added. The reaction flask was then filled with distilled water and mixed. The absorbance was measured at 510 nm. Total flavonoid compounds were calculated using a standard curve prepared with dilutions of an epicatechin standard, [38].

### 2.7. Statistical Analysis

A complete factorial experimental design, 2^2^ with 3 center points for a total of 11 experiments (Table 1), was applied to enable to evaluate of the influence of different concentrations of the antioxidant additives incorporated into the bio-based films. Palm oil carotenoid colorant (0.00 to 0.05%; *X_1_*) and green tea extract (0.00 to 5.00%; *X_2_*) were chosen as independent variables. The PI and TC from the packaged product (butter) and the TP, TF, TC, physical, barrier, mechanical and thermic properties from the films were used as dependent variables *(Y*). The data were subjected to variance analysis and Tukey’s test for comparison of means at a 5% significance level using Statistica 7.0 software (Minneapolis, USA).

**Table 1:** Coded and real values of green tea extracts and colorants added to cassava starch bio based films according to a (2^2^) second order experimental design with 3 central points.

FormulationFilms	Coded Values		Real Values (% w/w)	
	Colorant (X_1_)	Green tea extract (X_2_)	Colorant	Green tea extract
**F1**	−1.00	−1.00	0.01	0.70
**F2**	−1.00	1.00	0.01	4.30
**F3**	1.00	−1.00	0.04	0.70
**F4**	1.00	1.00	0.04	4.30
**F5**	−1.41	0.00	0.00	2.50
**F6**	1.41	0.00	0.05	2.50
**F7**	0.00	−1.41	0.03	0.00
**F8**	0.00	1.41	0.03	5.00
**F9(c)**	0.00	0.00	0.03	2.50
**F10(c)**	0.00	0.00	0.03	2.50
**F11(c)**	0.00	0.00	0.03	2.50

(c) Central points.

A quadratic regression model was employed to predict each response:
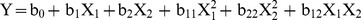
(3)where *Y* are the predicted responses, *X_1_* and *X_2_* are the independent variables, *b_0_* is the offset term, *b_1_* and *b_2_* are the linear effects, *b_11_* and *b_22_* are the squared effects and *b_12_* is the interaction term.

The goodness of fit of the models was evaluated by the determination coefficient (R^2^), an analysis of variance (ANOVA) and Fischer’s t test.

## Results and Discussion

### 3.1. Characterization of the Films

Table 2 shows the results of the thickness (t), water vapor permeability (WVP), mechanical properties (tensile strength and percent elongation at break), and thermogravimetric data of TGA (mass loss) and DTG (rate of mass loss), of the 11 film formulations, as responses (*Y*) according to a 2^2^ central composite experimental design with 3 central points.

**Table 2:** Thickness (t), mechanical properties (TS and ε), water vapor permeability (WVP), and thermal analysis (thermic events) of cassava starch films with addition of carotenoid colorant and green tea extract according to a (2^2^) second order experimental design with 3 central points.

Formulation Films	Real Values		t (mm)	TS (MPa)	ε (%)	WVP × 10^−8^(g_H2O_.mm/m^2^.h.kPa)	1^st^ event		2^nd^event	
	Carotenoic Colorante (*X_1_*, %)	Green Tea Extract (*X_2_*, %)					T _onset_ (°C)	Mass loss (%)	T _onset_ (°C)	Mass loss (%)
**C2**	–	–	0.204±0.015^a^	0.83±0.22^c^	253.30±0.05^a^	15.86±0.18^a^	38.72^c^	8.87^d^	315.23 ^a^	81.43^d^
**F1**	0.01	0.70	0.177±0.021^b^	2.45±0.18^d^	66.31±0.07^b^	2.94±0.19^b^	31.55^a^	13.12^a^	323.58^e^	66.87^a^
**F2**	0.01	4.30	0.212±0.012^c^	0.73±0.10^e^	90.61±0.08^a^	2.95±0.13^b^	29.35^d^	13.63^a^	301.10^b^	66.06^b^
**F3**	0.04	0.70	0.165±0.005^d^	1.71±0.30^f^	78.21±0.17^b^	5.65±0.30^c^	33.16^e^	11.79^b^	327.41^f^	70.65^e^
**F4**	0.04	4.30	0.196±0.010^e^	0.96±0.22^g^	131.10±0.11^a^	0.29±0.93^d^	39.26^f^	12.91^a^	301.97^b.c^	65.13 ^c^
**F5**	0.00	2.50	0.199±0.015^f^	1.66±0.07^h^	62.59±0.06^b^	2.05±0.65^e^	26.50^g^	13.06^a^	308.92^g^	72.93^f^
**F6**	0.05	2.50	0.193±0.012^g^	1.16±0.17^a^	105.80±0.13^a^	0.68±0.12^f^	32.04^b^	13.12^a^	313.93^a^	64.94^a^
**F7**	0.03	0.00	0.164±0.012^h^	4.36±0.58^i^	40.33±1.06^c^	7.60±0.19^g^	29.82^d^	11.27^b,c^	334.45^h^	76.62^g^
**F8**	0.03	5.00	0.193±0.01^i^	1.55±0.28^b^	84.54±3.17^a^	8.56±0.47^h^	46.68^h^	11.83^b^	298.14^i^	66.59^a,b^
**F9 (c)**	0.03	2.50	0.202±0.027^j^	1.58±0.24^b^	142.10±0.21^a^	6.43±0.20^i^	31.84^a,b^	11.12^b^	303.13^c^	55.54^c^
**F10 (c)**	0.03	2.50	0.200±0.07^l^	1.40±0.30^j^	157.00±0.22^a^	6.51±0.12^f^	32.26^b^	10.92^c^	306.78^d^	55.01^c^
**F11 (c)**	0.03	2.50	0.210±0.02^m^	1.12±0.4^a^	126.90±0.30^a^	7.63±0.85^g^	31.64^b^	11.03^c^	305.93^d^	54.94^c^

Control C2 = additives without film; (c) Central points. TS = Tensile strength (MPa); ε = percent elongation at break (%); T_onset_ = degradation onset temperature (°C). Means with the same letters in the same columns were not statistically different (p>0.05) according to Tukey’s test.

The thickness of the starch films with different quantities of two additives varied of 0.164 to 0.212 mm and was 24.39% higher and 9.92% smaller when compared to control film, (C2, Table 2). Control of the thickness in the films produced by casting is a step that requires great attention since variations here can affect their properties, including the mechanical and barrier properties, which certainly compromise the performance of the package [39], [40].

The incorporation of two additives into cassava bio-based films caused significant difference (p>0.05) between thickness of the 11 formulations and those in relation C2 control, according to Tukey’s test (Table 2). For this parameters, ANOVA also indicated that the differences between the formulations were not statistically significant (p>0.05).

#### 3.1.1. Mechanical Properties

The effect of the extract concentration on the mechanical properties was evaluated by the tensile strength (TS) and elongation at the break percentage (ε) of the films. For all films, the values of TS varied from 0.730±0.10 to 4.360±0.58 MPa, and the ε varied from 40.33±1.06 to 157.00±0.22% (Table 2).

A comparison between TS of the films containing additives with C2 control film showed greater tensile strength in all formulations, except F2. However, all 11 formulations showed lower percent elongation at break (**ε**) than C2 control (Table 2).

The formulation F7 (0.03% of carotenoic colorant and 0.00% of green tea extract) showed a higher TS and a lower percent elongation at break (Table 2), which characterized this material as more rigid than the control. These differences on the mechanical behavior of the formulated films could be explained by the carotenoic colorant interacting with films constituents and changing the properties of the continuous phases and the effect of crystallinity formed after the processing and storage of starch film. Furthermore, the molecule regularity of amylose provided for the formation of crystalline regions in formulation F7 and, together with a greater number of points of contact, contributed to a behavior similar to conventional semi-crystalline polymers, for instance, a higher tensile strength and lower elongation at break percentage.

Between the formulations with 0.03% carotenoic colorant, the formulations F10, F11 and F12 (central points) were less affected in elongation at break percentage by comparison with control (Table 2).

**Figure 1:**
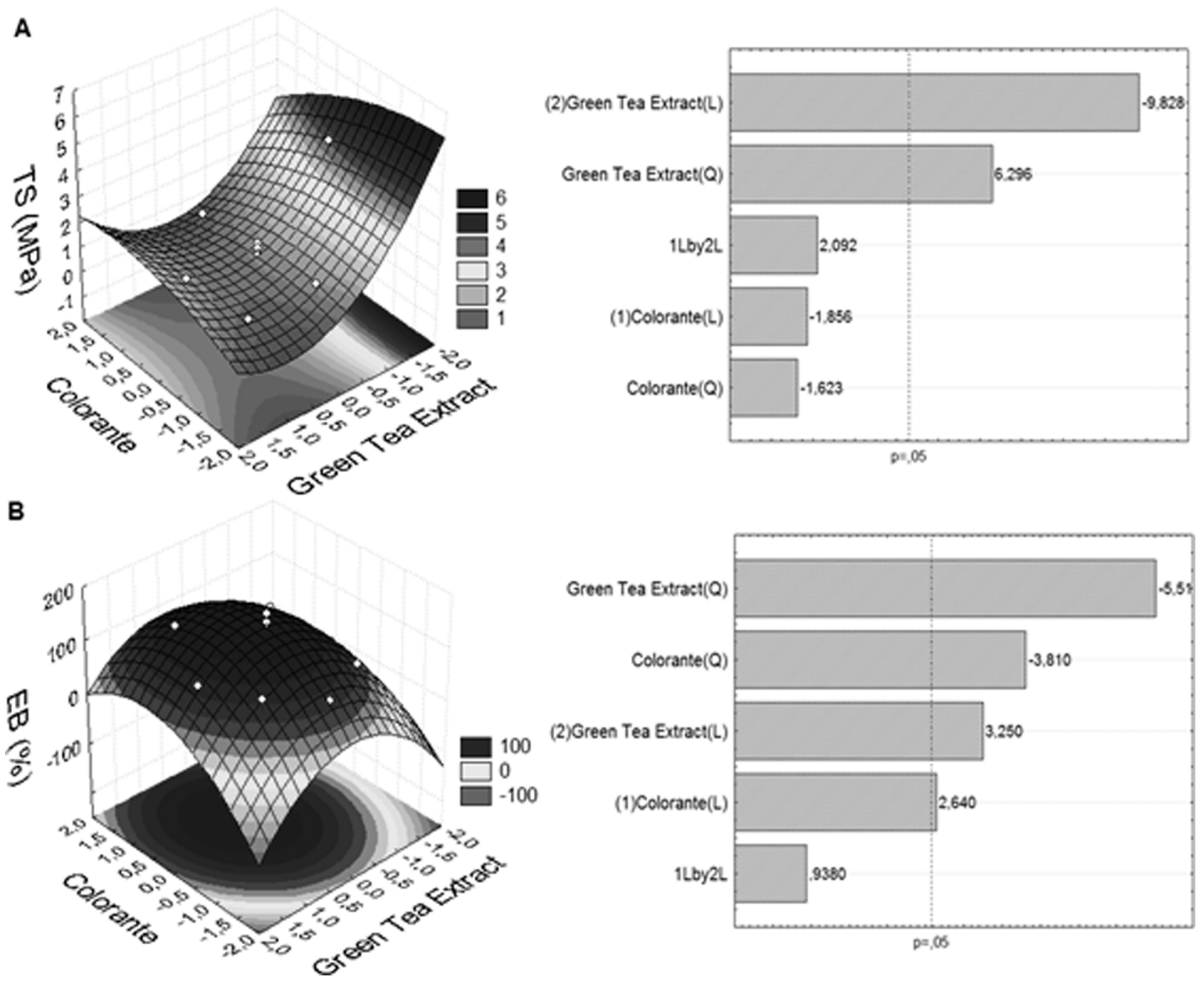
Response surface plot and Pareto chart for understanding the tensile strength (TS) and elongation at break percentage (EB) (*Y* response), as function on the content of carotenoic colorant and green tea extract (coded values of *X_1_* e *X_2_*).

The incorporation of additives in certain concentrations tested confer greater resistance to films (Table 2), an important characteristic for use in the packaging sector in general, where large deformation (ε) of the films is not required. Figure 1 shows the response surface and Pareto chart to tensile strength (Fig. 1A) and elongation at break percentage (Fig. 1A).

The ANOVA statistical analysis applied to the results indicated that the addition of green tea extract (X_2_) negatively affected the tensile strength, while both additives (X_1_ and X_2_) the elongation at break percentage (Fig. 1).

The properties of the starch films depends a series factors, such as, the nature of starch and its cohesive structure, type of processing, environmental conditions, type of plasticizer, their thickness among others [39].

The film base already had added plasticizers (glycerol), and the concentration in the final material may have been too high, resulting in excessive interactions between the film network and the plasticizers and lower film flexibility. This variation could be caused by some of the natural compounds present in the extracts, which could greatly affect a starch film network and the mechanical performance [40], [41].

The humidifying ability of such components can alter the mechanical resistance of the biodegradable materials. Natural components that absorb water can increase the hydrophilicity of cassava starch biobased films, which are already highly hydrophilic materials, as function the type of processing and the environmental conditions of storage [42].

Correlation inversely proportional of the thickness (0.164–0.212 mm) with the tensile strength (0.73–4.36 MPa) and break elongation (40.33–157.00%) (Table 2), of r^2^ = 0.594 and r^2^ = 0.391 respectively, indicate that this parameter can exert influence on the mechanical properties of the films analyzed. Janson and Thuvander [43] also observed same effect, a decrease from 100 to 20% in elongation with increasing thickness 0.3 to 2.5 mm. According to authors, the effect in mechanical resistance could be too explained by the difference in the thickness among the samples, although it was also not statistically significant (p>0.05).

#### 3.1.2. Water Vapor Permeability

The water vapor permeability coefficient of a film is a constant value for permeation of the water vapor at a given temperature. The permeability of a film depends on the chemical structure and morphology, the nature of permeate and the temperature of the environment [44]. The water vapor permeability of the cassava starch bio-based films incorporated with different concentrations of carotenoic colorant and green tea was examined.

**Figure 2:**
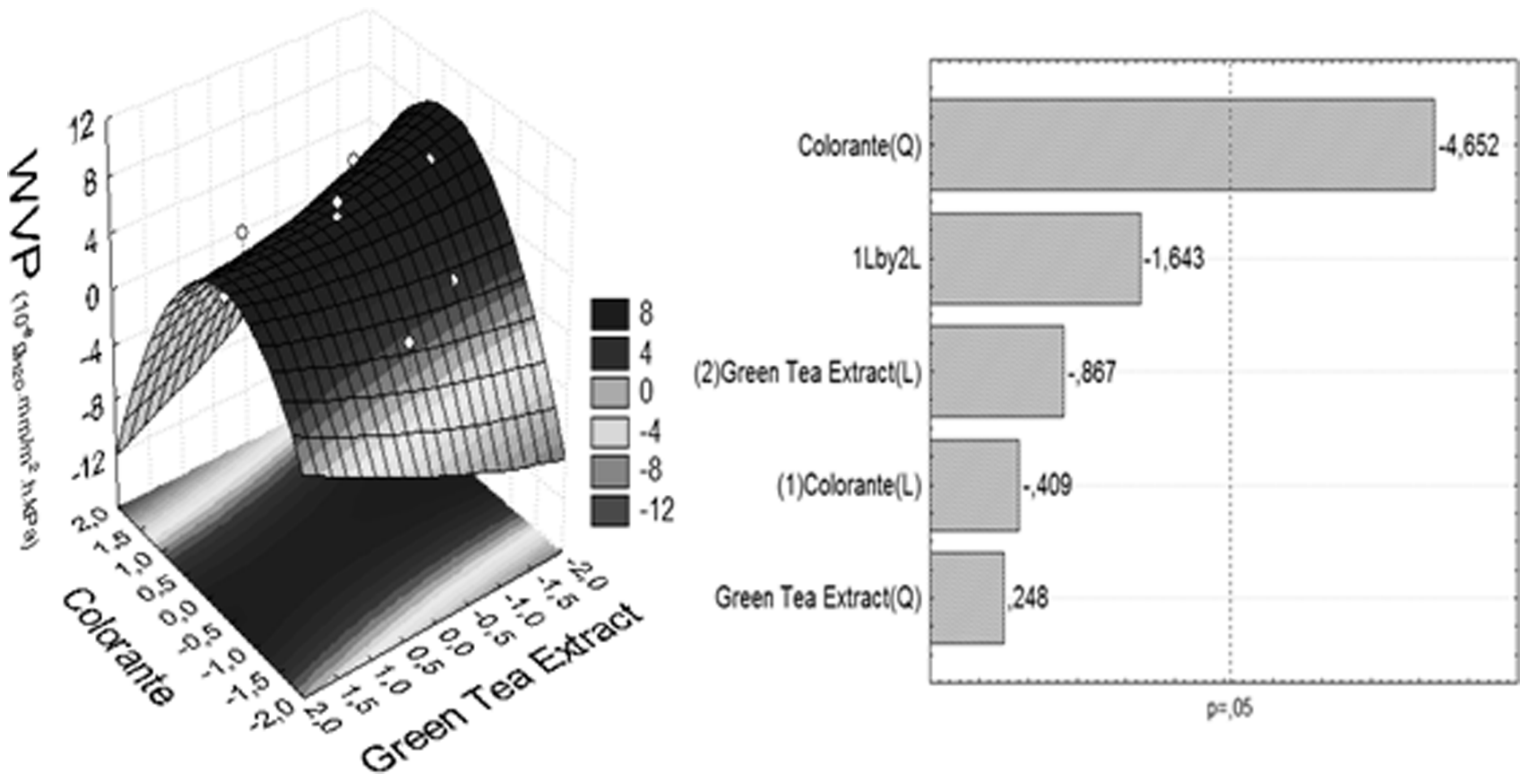
Response surface plot and Pareto chart for understanding water vapor permeability (WVP), as function on the content of colorant and GTE, with additives coded values (*X_1_* e *X_2_*).

The data indicate (Table 2) that the incorporation of additives resulted a decrease of 50 times in water vapor permeability when compared to C2 control (additives without film). The incorporation of additives, (independent variables) in different percentages in cassava starch plasticized with glycerol matrix resulted in favorable statistically significant effect (p<0.05) in WVP (Table 2; Fig. 2). These results can be explained mainly by the nature of the carotenoic colorant and the films constituents, which can give increased cohesion in the matrix.

The lower WVP of the films incorporated with additives might result from the interactions of mainly of carotenoic compounds with hydrophobic regions of leached amylose and with amylopectin side chains through Van der Waals forces, which limit the availability of hydrogen groups to form hydrophilic bonding with water [45]. This leads to a decrease in the affinity of the cassava starch film towards water.

Although the effect of incorporation of green tea extract was not significant, may there is a contribution of its components to the reduction of WVP. Siripatrawan and Harte [17] reported the influence the green tea extracts in chitosan films. The permeability coefficient decreased from 0.256±0.023 to 0.087±0.012 g mm m^−2^ d^−1^ kPa^−1^, while the density increased from 1.21±0.03 to 1.67±0.03 g cm^−3^, as the concentration of green tea increased from 0 to 20%. Incorporation of green tea into chitosan film caused the resulting films to become denser, with less water vapor permeability. Curcio and others [46] also observed the formation of covalent bonds between gallic acid antioxidant and chitosan, as verified by FTIR.

#### 3.1.3. Thermogravimetric analyze

TG is a technique in which the change in mass of the sample is determined by the temperature and/or time [47]. Schlemmer investigated the behavior of pure cassava starch by TGA [48]. The starch had only one stage of decomposition, with a Td thermal decomposition value of approximately 300°C. As illustrated in Table 2, we observed only two events: a very slight weight reduction approximately 100°C, attributed to lost water, reducing the mass of the material at least 12%; and a second weight reduction at approximately 300°C, which corresponds to the degradation of the starch and the highest percentage of mass loss at approximately 70% in the formulated films and in the control films at 600°C.

**Figure 3:**
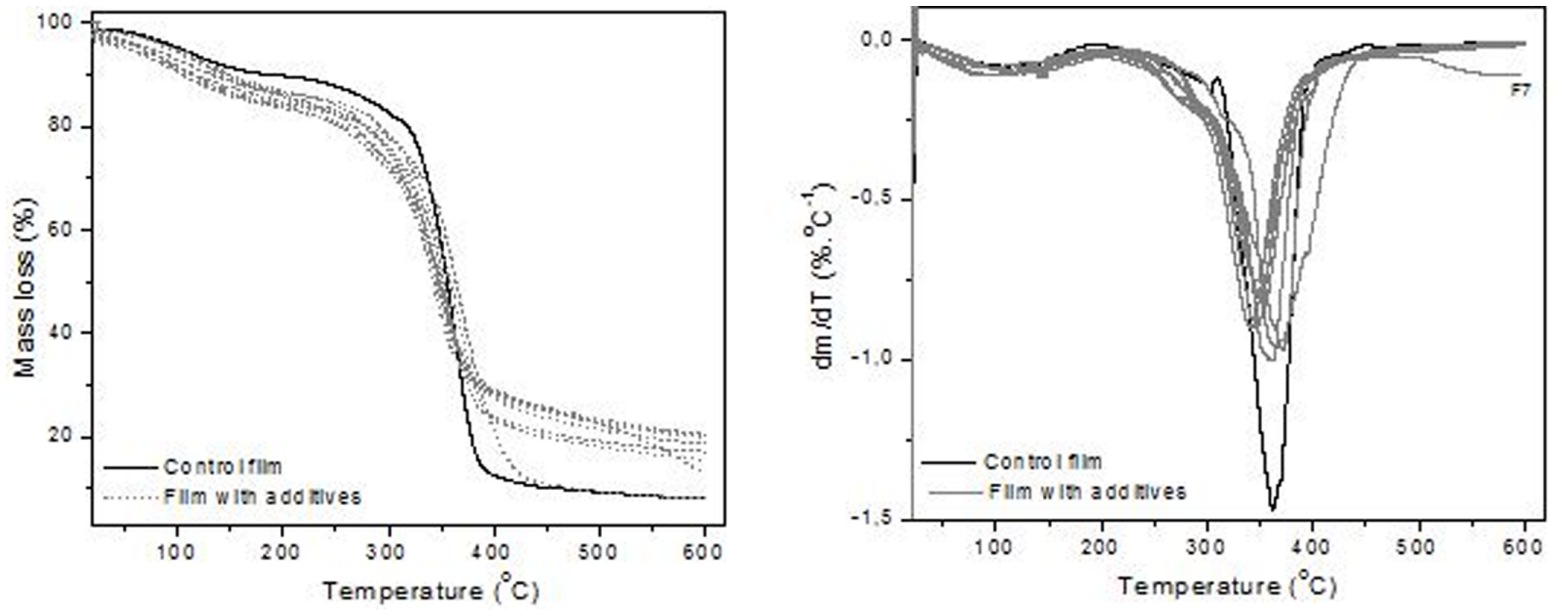
TG (A) and DTG (B) curves for the control film and the film with different additives concentrations.

The analysis facilitates the knowledge about possible interactions between the matrix and the additives by providing information about the stability and applicability of the developed film. Figures 3a e b presents the TG and DTG curves of the starch films. Despite some differences in terms of the presence of bound water and residual mass, similar profiles in the curves of mass loss were observed of the analyzed material presents with the pure starch and control film, indicating good interactions in the polymer matrix.

The percentage of bound water varies from 9% to 13% and the main stage of degradation, which corresponds to ∼70% of mass loss, starts at ∼ 200°C and the residual mass, is 15% at 600°C. It is noteworthy that the humidity of the films ranged from 7 to 10%. Apparently the residual mass is related to the nature of the additives, impurities and inorganic components, and the conditions for analysis in an inert atmosphere (N_2_), so there is no complete burning, even organic substances.

In the first event, when compared to the control, formulations with different concentrations of additives, showed a decrease in temperature beginning at 20%, except for the F8 formulation, and formulations with high concentrations of additives (F3, F4 and F8) this temperature increased by 10% and the weight loss was over 60% compared to the mass loss of control. In the latter event, there was a reduction T_onset_, Td_2_ and 5%. However for the formulation F7 (0.03% colorant), the behavior was different from the others, where T_onset_ concerning the decomposition event was shifted to higher temperature suggesting greater interaction between the additive and the constituents of polymer matrix. The formulation F7 (0.03% CCN) when compared to control and other formulations in different parameters analyzed always showed characteristics that confirmed the cohesiveness of the matrix under these conditions.

Some discrepancies can also be observed in the method thermometric, as function probably due to small changes in mass of the samples used in the analysis and the high sensibility.

### 3.2. Stability of Additives Incorporated into Bio-based Films during the Storage of the Packaged Product

The green tea extract and colorant were incorporated in films of cassava starch as a source of active compounds, as evidenced in other studies, carotenoids and phenolic compounds are the two major groups of bioactive compounds with antioxidant activity [36], [49], [50]. According to the experimental design, different concentrations of these compounds were incorporated into the polymer matrix, and these compounds in films were monitoring as total carotenoids (TC), total polyphenols (PT) and flavonoids (TF) during storage of the packaged butter.

All the parameters evaluated in the films (TP, TF and TC) showed changes during the storage of the packaged product for 45 days, suffering significant reductions, the storage conditions can be attributed to migration to packaged product by oxidation and decomposition, can protect the packaged product (Table 3).

**Table 3:** Decrease of antioxidants in the film formulations with different concentrations of green tea extracts and carotenoic colorant after butter storage by 45 days.

Formulation Films	Real Values		Decrease TP (mg/100 g)	Decrease TF (mg/g)	Decrease TC (µg/g)
	Colorante (%)	Green Tea Extract (%)			
**F1**	0.01	0.70	17.10	16.43	0.55
**F2**	0.01	4.30	33.72	35.69	1.95
**F3**	0.04	0.70	17.89	5.55	11.44
**F4**	0.04	4.30	22.03	36.82	11.63
**F5**	0.00	2.50	26.24	20.00	0.83
**F6**	0.05	2.50	49.11	28.24	13.32
**F7**	0.03	0.00	0.44	0.49	6.59
**F8**	0.03	5.00	28.60	20.28	2.88
**F9(c)**	0.03	2.50	19.06	37.17	3.38
**F10(c)**	0.03	2.50	19.41	31.34	2.53
**F11(c)**	0.03	2.50	25.40	34.95	3.98

(c): Central Points. TC: Total Carotenoids. TP: Total Polyphenols. TF: Total Flavonoids.

The formulations films showed reductions in levels of TP, TF and TC ranging from 11.10 to 53.00%, from 14.61 to 68.56% and from 11.77 to 91.52%, respectively (Table 3), demonstrating that even after 45 days of storage, part of the bioactive compounds of additions remained viable in the films. It is observed that the formulation F5 (only green tea extract) showed the greatest reduction in the content of TC (0.007 mg/g, 91.51%) of the formulation F7 (only carotenoic colorant) (9.84 mg/g 40.09%), probably due to the stability of the pigment found in colorant compared to the extract, whereas for the content of polyphenols and flavonoids for the F7 formulation showed the greatest reductions in baseline (53.00% and 68.56%).

The formulations of the central points, on average, showed the lowest reduction in the content of TP (11.63%) followed by F6 (28.30%). The formulation F8 showed a smaller reduction in the content of TF (14.61%) and F1 showed high reduction (42.82%). The content of TC, F1 showed a lower reduction (11.76%) and F3 had the second highest (41.67%) of the initial content after 45 days of storage of the packed product (Table 3).

A similar behavior was observed in films containing mango and acerola pulps added as antioxidants, which were used to pack palm oil. In this case, a decrease after 45 days of storage ranged from 24.53 to 43.60% for TC, while decreases in polyphenols and vitamin C ranged from 17.80 to 36.12% and from 69.50 to 85.00%, respectively [8].

According to Wessling and others [13] tocopherol incorporated in polyethylene materials showed a resistance during 4 weeks of storage than those film controls containing butylated hydroxytoluene (BHT), which degraded in just in 1 week.

**Figure 4:**
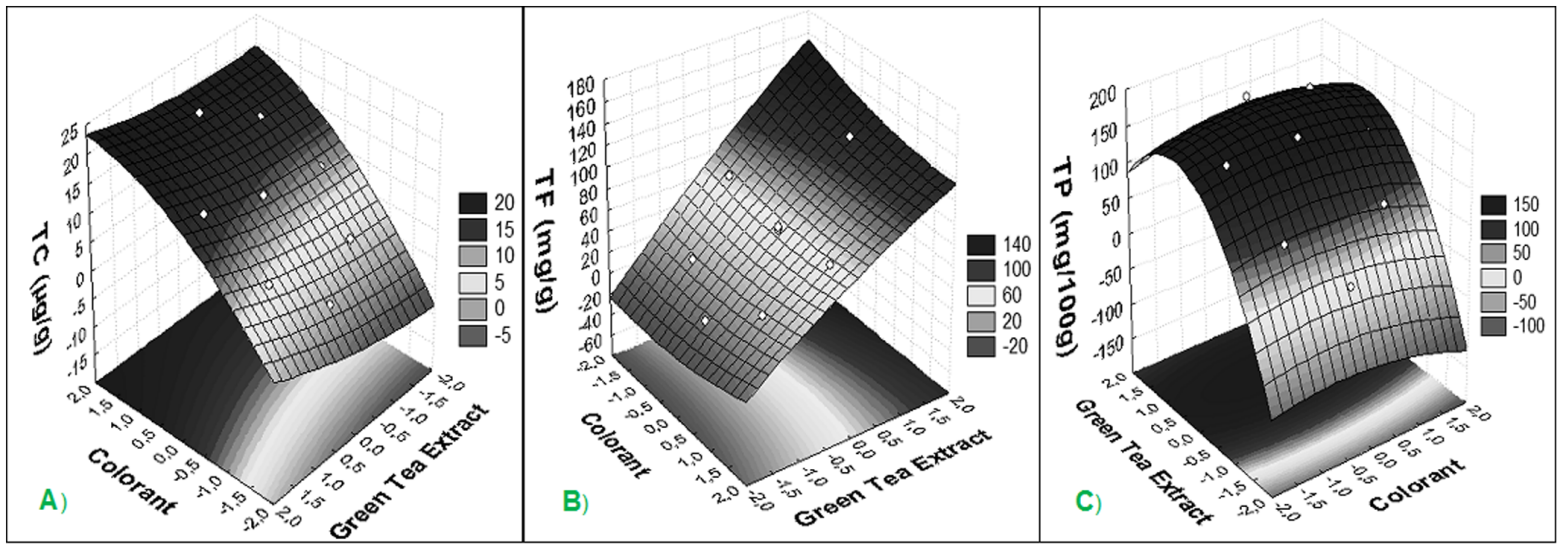
The response surface plot demonstrating the effect of incorporated additives to illustrate the effect of the increased factor on TP (mg/100 g), TF (mg/g) and TC (µg/g) film preparation after 45 days of butter storage.

Experimental results for the different formulations of films used to package butter showed significant (p<0.05) differences in TP, TF and TC after 45 days of storage (Fig. 4). This resulted in a second-order polynomial equation, which represents the model equation used to evaluate the increase of TP (eq. 4) in films as a function of the concentrations of colorant (%, X_1_) and green tea extract (%, X_2_) and the interaction between them (X_1_ and X_2_). According to eq. 4, the increase in the TP concentration in films depends upon the interaction between both independent variables (X_1_ and X_2_), while the decrease in the TP concentration only depends upon these variables independently.
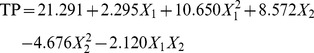
(4)


R^2^ = 0.88.



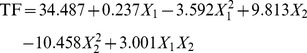
(5)


R^2^ = 0.86.



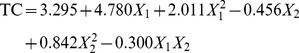
(6)


R^2^ = 0.96.

### 3.3. Monitoring Packaged butter during Storage

As expected, the butter packaged in different formulations of biodegradable films incorporated with different concentrations of carotenoic colorant and green tea extract (F1 through F11) showed a change in the initial content of total carotenoids and an differentiate increase in the peroxide index. Butter packaged in the active films had a smaller increase in PI (p<0.05) when compared with the three controls, the peroxide index increased 692.00% in exposed butter (C1, unpackaged), 559.21% in packaged formulation without additives (C2) and 583.73% in LDPE (C3), indicating the effectiveness of the additives as antioxidants (Table 4). This effect appears to be dependent on the additives concentrations because the butter packed in films (F8, F2 and F4 - high concentrations of green tea extract) had a greater oxidation (PI 4.330, 4.307 and 4.221 meq/kg; 456.67, 451.45 e 451.14%) compared to F6-butter (average concentration of additives) (PI 2.576 meq/kg; 231.57%) (p<0.05), under the same storage conditions after 45 days (Table 4).

**Table 4:** Alterations of the butter packed in different film formulations after 7, 15, 30, and 45 days of storage.

Formulation Films	Real Values		Increase PI (%)				TC, Increase (+), decrease (−), (%)
	Colorante (%)	Green Tea Extract (%)	0–7	0–15	0–30	0–45	0–45
**C1**	–	–	271.1	424.9	474.4	692.1	−12.01
**C2**	–	–	208.1	327.8	379.3	559.2	−3.72
**C3**	–	–	244.1	374.6	442.8	583.7	−15.13
**F1**	0.01	0.70	90.4	204.2	286.8	365.4	+60.00
**F2**	0.01	4.30	96.2	261.6	315.7	451.4	+41.62
**F3**	0.04	0.70	79.6	184.2	261.0	328.2	+111.90
**F4**	0.04	4.30	96.4	227.9	305.2	451.1	+86.51
**F5**	0.00	2.50	79.4	147.1	253.4	330.3	−15.22
**F6**	0.05	2.50	38.5	102.3	205.6	231.6	+154.91
**F7**	0.03	0.00	88.9	209.1	295.1	412.3	+66.00
**F8**	0.03	5.00	113.4	258.9	342.3	456.7	+111.80
**F9(c)**	0.03	2.50	81.2	150.7	258.9	319.3	+90.61
**F10(c)**	0.03	2.50	76.6	134.3	236.5	284.8	+78.70
**F11(c)**	0.03	2.50	77.6	133.9	241.9	297.1	+109.90

(c) Central points. C1 = unpackaged; C2 = starch films without additives, C3 = package in LPDE.

The peroxide index (PI) of the butter packaged in formulation F8 (0.03% colorant and 5% green tea extract) increased in 456.67% after 45 days of storage, while the butter packed in the formulation F6 (0.05% colorant and 2.5% green tea extract) showed lower PI (231.57%) (p<0.05), demonstrating that as increase the concentration of active compounds incorporated in packaging, enhances the oxidation of the packed product (Table 4).

The butter packed in film formulation F7 (only colorant) had a higher PI (PI = 3.980 meq/kg; 412.27%) than butter packed in formulation F5 (only green tea extract) (PI = 3.294 meq/kg; 330.34%). This demonstrates that a greater protective effect of phenolic compounds exists, and that the green tea extract was more effective than the colorant (p<0.05) when used individually (Table 4).

The packaged butter showed an increase in total carotenoids values after 45 days of storage, that was proportional to the concentration of colorant incorporated in the films (r^2^ = 0.87). For example, butter packed in F6 (maximum concentration of colorant) showed greater increase in TC (154.9%) over the butter contained in the formulations F1 and F2 (0.01% only colorant) of 60 and 41%, respectively, under the same storage conditions.

**Figure 5:**
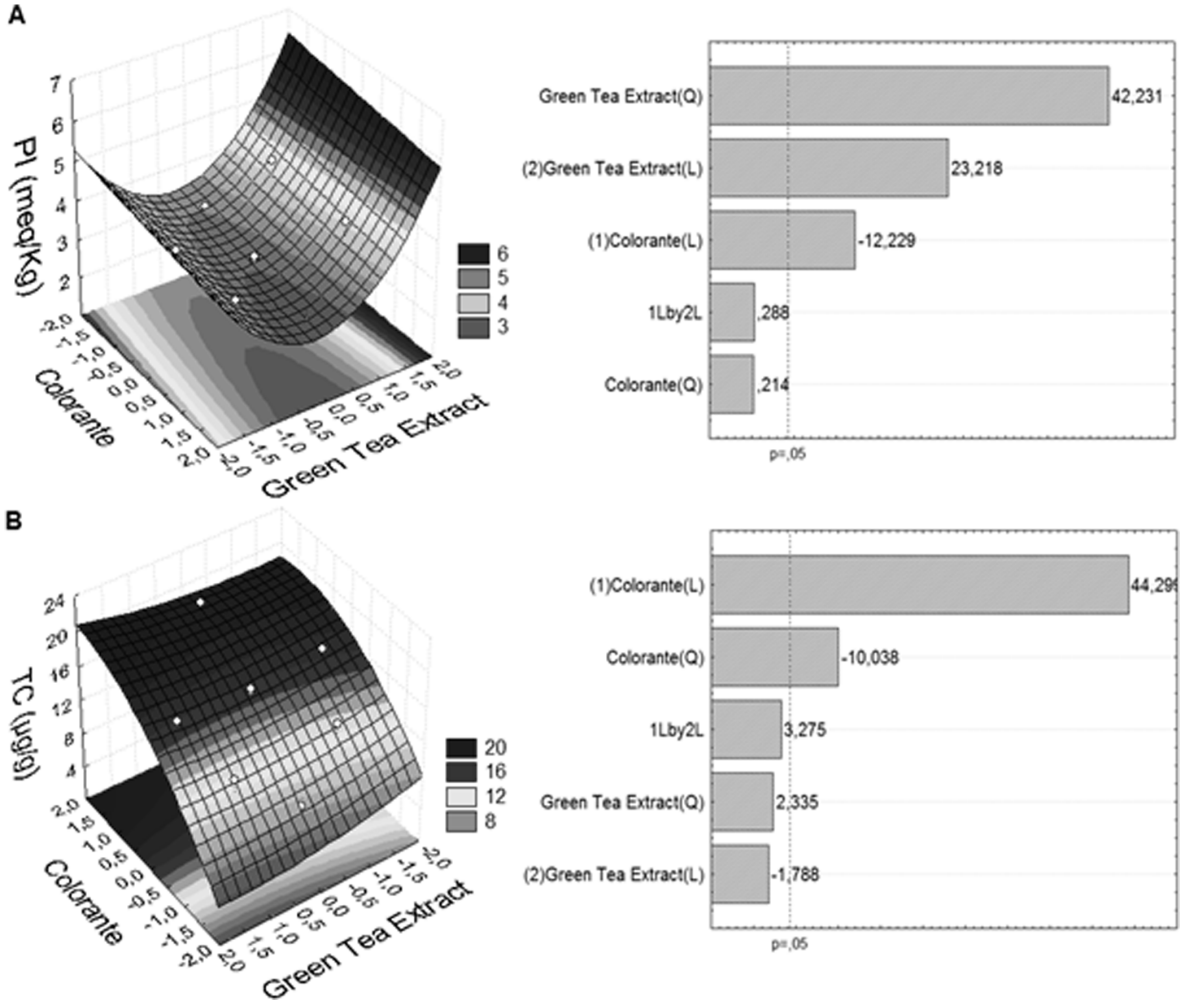
Response surface plot and Pareto chart for understanding the increase of peroxide index (PI) values and total carotenoids (TC) after 45 days of butter storage.

In this context, the colorant (%, X_1_) and green tea extract (%, X_2_) incorporated into the films in different proportions, and increase in the PI content (meq/kg) of butter after 45 days of storage can be expressed using a second-order polynomial equation (eq. 7). The increase in this parameter depends upon the concentration of the colorant and green tea extract and the interaction of these two factors. Figure 5 shows the response surface graph, illustrating the increase of butter PI (Fig. 5A) and TC (Fig. 5B) packaged with different film formulations. The graph indicates that the point of maximum PI increase corresponds to the maximum concentration of both additives, and the point of minimum PI occurs at the average concentration of both additives (p<0.05).

The Pareto chart of standardized effect estimates (absolute values) showed the magnitude of the positive effect of green tea extract concentration (quadratic and linear function) on increasing PI, demonstrating that depending on concentration, this additive has a favorable influence on that variable. However, for the day 45, the Pareto chart demonstrated that the linear function of the colorant variable showed a negative effect on the increase in PI, (Fig. 5A).
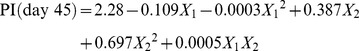
(7)


R^2^ = 0.98.
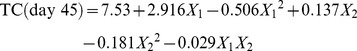
(8)


R^2^ = 0.86.

In equation 8, it is verified that the carotenoid content of the butter only depends on the linear term of the colorant concentration (p<0.05) with a correlation coefficient of 86%, obtaining a first order model, which is suitable for describe the results shown in the response surface. This demonstrates the migration of carotenoid packaging for butter. This result was desired, as for the mechanism of active packaging is efficient active compound must migrate to the packaged product thus exerting the desired action.

These results are in agreement with some studies that have shown that oxygen can permeate through the film, reacting preferentially with some compounds present in the film formulation. This allows the packaged product to be preserved for a longer period of time [16]. Justifying the incorporation of antioxidant additives in packaging, the colorant and green tea extract, as additives in film-forming dispersions, are effective in preserving a packaged product against oxidation.

### 3.4. Correlations between parameters of the Bio-based Films and Packing Butter during Storage

The results of this study suggest that the protection of packaged products against oxidation can be attributed to the concentration dependent radical scavenging activity of antioxidant compounds present in film forming dispersions.

Considering the results for the PI values from the packaged product, the protective effect against lipid oxidation is likely a consequence of a physical process because of the carotenoids content. This is especially true of the colorant because the butter packed in films containing this additive showed a lower rate of oxidation compared to the controls.

When comparing the results of the 11 formulations, it appears that the butter wrapped in the formulations that had higher levels of green tea had a significant increase in peroxide index (about 450%, p<0.05). This result is likely a result of pro-oxidant actions of the bioactive compounds (Table 4), as other studies have reported that high concentrations of these compounds can result in pro-oxidant activity under certain conditions [49], [50] when there is an imbalance between oxidant and antioxidant molecules, which results in the induction of oxidative damage by free radicals, or if there is no equivalent concentration of the other antioxidants to regenerate the radical. These oxidative stress mechanisms are not yet understood [49], [50], [51], [52].

### 3.5. Films Applications

The applications of starch-based plastics can include biodegradable packaging in the food industry and pharmaceutical, particularly as coatings to enhance the shelf life of fatty products [53], [54], [55]. The commercial application of films containing food starch depends of improvements on the mechanical properties and the water vapor permeability of these materials, which are limiting properties [53]. Researches are needed to proposing improvements, development new products by combination this biopolymer with new materials, biopolymeric blends and composites, as biodegradable nanoparticles, for to improve coating properties and increase potential applications of food packaging [1], [53], [55], [56]. Biodegradable films can be applied to food products, such as a barrier for fat, butter and margarine [54], as active biodegradable films that can protect the packaged product. The functionality of the product is related also to the how well the food product resists deterioration.

## Conclusions

This study indicated that an active film from cassava starch films could be achieved by incorporation with colorant and green tea extract, as a source of bioactive compounds, into a cassava starch packaging for lipid foods. Addition of colorant and green tea extract improved significantly functional properties mechanical, water vapor barrier and antioxidant properties of the resulting films. These changes, as verified by results, could be attributed to the interactions between functional groups of starch, colorant and green tea extract polyphenol compounds, cohesive molecular reorganization, which resulted in a reduction in permeability to water vapor and providing greater rigidity to the material compared to the controls. The results provide an oxidative protection in packaged butter, by decrease peroxide index, when using these film additives. However, high colorant and green tea contents can act as pro-oxidant agents, which suggest that these additives should be used at low concentrations. Nevertheless, further studies are required before using this film as an active packaging for food products, to verify this activity and the effects of incorporating antioxidant additives into other matrixes.

## References

[1] Khwaldia K, Arab-Tehrany E, Desobry S (2010) Biopolymer coatings on paper packaging materials. Comprehensive Reviews in Food Science and Food Safety 9: 82–91. 10.1111/j.1541-4337.2009.00095.x 33467805

[2] Moradi M, Tajik H, Rohani SMR, Oromiehie AR, Malekinejad H, et al (2012) Characterization of antioxidant chitosan film incorporated with *Zataria multiflora* Boiss essential oil and grape seed extract. Food Science Technology 46: 477–84.

[3] Vieira MGA, da Silva MA, Santos LO, Beppu MM (2011) Natural-based plasticizers and biopolymer films: A review. European Polymer Journal 47: 254–63.

[4] Bonilla J, Atarés L, Vargas M, Chiralt A (2013) Properties of wheat starch film-forming dispersions and films as affected by chitosan addition. Journal Food Engineering 114: 301–12.

[5] FAOSTAT - Food and Agriculture Organization of The United Nations, Production Crops FAO Codes (2012) http://faostat.fao.org/site/567/DesktopDefault.aspx?PageID=567.

[6] Gimenez B, Lacey AL, Pérez-Santín E, López-Caballero ME, Montero P (2013) Release of active compounds from agar and agar-gelatin films with green tea extract. Food Hydrocolloids 30: 264–71.

[7] Vermeiren L, Devlieghere F, Van Beest M, de Kruijf N, Debevere J (1999) Developments in the active packaging of foods. Trends Food Science Technology 10: 77–86.

[8] Souza CO, Silva LT, Silva JR, Lopez JA, Veiga-Santos P, et al (2011) Mango and acerola pulps as antioxidant additives in cassava starch bio-based film. Journal Agricultural Food Chemistry 59: 2248–54. 10.1021/jf1040405 21361289

[9] Falguera V, Quintera JP, Jimenez A, Munoz JA, Ibarz A (2011) Edible films and coatings: structures, active functions and trends in their use Trends. Food Science Technology 22: 292–303.

[10] Miller KS, Krochta JM (1997) Oxygen and aroma barrier properties of edible films: A review. Trends Food Science Technology 8: 228–37.

[11] Finley JW, Given Jr P (1986) Technological necessity of antioxidants in the food industry. Food Chemical Toxicology. 24: 999–1006. 10.1016/0278-6915(86)90280-2 3804129

[12] Nerín C, Tovar L, Salafranca J (2008) Behaviour of a new antioxidant active film versus oxidizable model compounds. Journal Food Engineering 84: 313–20.

[13] Wessling C, Nielsen T, Andres L (2000) The influence of atocopherol concentration on the stability of linoleic acid and the properties of low-density polyethylene. Packaging Technology and Science 13: 19–28.

[14] Heumann BF (1990) Antioxidants: Firms seeking products they can label as ‘natural’. INFORM. 1(12) http://aocs.files.cms-plus.com/inform/1990/12/1002.pdf.

[15] Song HS, Bae JK, Park I (2013) Effect of heating on DPPH radical scavenging activity of meat substitute. Preventive Nutrition and Food Science 18(1): 80–84. 24471114 10.3746/pnf.2013.18.1.080 PMC3867152

[16] Grisi CVB, Veiga-Santos P, Silva LT, Cabral-Albuquerque EC, Druzian JI (2008) Evaluation of the viability of incorporating natural antioxidants in bio-based packagings. In Food Chemistry Research Developments; Nova Science Publishers 1: 1–11.

[17] Siripatrawan U, Harte BR (2010) Physical properties and antioxidant activity of an active film from chitosan incorporated with green tea extract. Food Hydrocolloids 24: 770–5.

[18] Giménez B, Moreno S, López-Caballero ME, Montero P, Gómez-Guillén MC (2013) Antioxidant properties of green tea extract incorporated to fish gelatin films after simulated gastrointestinal enzymatic digestion. LWT - Food Science and Technology 53(2): 445–451.

[19] Martín-Diana AB, Rico D, Barry-Ryan C (2008) Green tea extract as a natural antioxidant to extend the shelf-life of fresh-cut lettuce. Innovative Food Science and Emerging Technologies 9: 593–603.

[20] He Y, Shahidi F (1997) Antioxidant activity of green tea and its catechins in a fish meat model system. Journal Agricultural Food Chemistry 45: 4262–6.

[21] Anesini C, Ferraro GE, Filip R (2008) Total polyphenol content and antioxidant capacity of commercially available tea (*Camellia sinensis*) in Argentina. Journal Agricultural Food Chemistry 56: 9225–9. 10.1021/jf8022782 18778031

[22] Gramza A, Khokharb S, Yokob S, Gliszczynska-Swigloc A, Hesa M, et al (2006) Antioxidant activity of tea extracts in lipids and correlation with polyphenol content. European Journal of Lipid Science and Technology 108: 351–62.

[23] Wanasundara UN, Shahidi F (1998) Antioxidant and pro-oxidant activity of green tea extracts in marine oils. Food Chemistry 63: 335–42.

[24] Rice-Evans CA, Miller NJ, Paganga G (1996) Structure-antioxidant activity relationships of flavonoids and phenolic acids. Free Radical Biology and Medicine 20: 933–956. 8743980 10.1016/0891-5849(95)02227-9

[25] Re R, Pellegrini N, Proteggente A, Pannala A, Yang M, et al (1999) Antioxidant activity applying an improved ABTS radical cation decolorization assay. Free Radical Biology and Medicine 26: 1231–7. 10381194 10.1016/s0891-5849(98)00315-3

[26] Montenegro MA, Rios AO, Mercadante AZ, Nazareno MA, Borsarelli CD (2004) Model studies on the photosensitized isomerization of bixin. J Agric Food Chem 52: 367–73. 14733523 10.1021/jf0349026

[27] Boon CM, Ng MH, Choo YM, Mok SL (2013) Super, red palm, and palm oleins improve the blood pressure, heart size, aortic media thickness and lipid profile in spontaneously hypertensive rats. Plos One 8: 2, e55908. 10.1371/journal.pone.0055908 PMC3569425 23409085

[28] Montenegro MA, Nunes IL, Mercadante AZ, Borsarelli CD (2007) Photoprotection of vitamins in skimmed milk by an aqueous soluble lycopene-gum arabic microcapsule. Journal Agricultural Food Chemistry 55: 323–9. 10.1021/jf0622883 17227061

[29] Azeredo HMC, Mattoso LHC, Wood D, Williams TG, Avena-Bustillos RJ, et al (2009) Nanocomposite edible films from mango Puree reinforced with cellulose Nanofibers. Journal Food Science 74: 31–5. 10.1111/j.1750-3841.2009.01186.x 19646052

[30] Nishiyama MF, Costa MF, Costa AM, Souza CG, Bôer CG, et al (2010) Brazilian green tea (*Camellia sinensis* var assamica): effect of infusion time, mode of packaging and preparation on the extraction efficiency of bioactive compounds and on the stability of the beverage. Food Science Technology 30: 191–6.

[31] ASTM Standards (1989) E96–80: Standard test methods for water vapor transmission of materials. West Conshohocken, Pennsylvania, USA.

[32] Veiga-Santos P, Suzuki CK, Cereda MP, Scamparini ARP (2007) Microstructure and color of starch-gum films: Effect of additives and deacetylated xanthan gum. Food Hydrocolloids 19: 1064–73.

[33] Gontard N, Gilbert S, Cuq JL (1993) Water and glycerol as plasticizer effect mechanical and water vapor barrier properties of an edible wheat gluten film. Journal Food Science 58: 206–11.

[34] ASTM - American Society for Testing and Materials (1995) Designation E96–95: Standard Method for Water Vapor Transmission of Materials. Philadelphia: ASTM. (Annual Book of ASTM Standards).

[35] Association of Official Analytical Chemists (2000) Official Methods of Analysis Cd 8b-90; AOAC: Gaithersburg, MD.

[36] Passoto JA, Penteado MVC, Mancini-Filho J (1998) Activity of β-carotene and vitamin A: A comparative study with synthetic antioxidant. Food Science Technology 18 3: 624–32.

[37] Silva SR, Mercadante AZ (2002) Carotenoid composition of fresh yellow passion fruit (*Passiflora edulis*). Food Science and Technology 22: 254–8.

[38] Lee ES, Lee HE, Shin JY, Yoon S, Moon JO (2003) The flavonoid quercetin inhibits dimethylnitrosamine-induced liver damage in rats. Journal Pharmacy Pharmacology 55(8): 1169–1174. 10.1211/0022357021396 12956909

[39] Xiong HG, Tang S, Tang H, Zou P (2008) The structure and properties of a starch-based biodegradable film. Carbohydrate Polymers 71 (2): 263–268.

[40] Jansson A, Thuvander F (2004) Influence of thickness on the mechanical properties for starch films. Carbohydrate Polymers 56: 499–503.

[41] Zhu F, et al (2009) Effect of phytochemical extracts on the pasting, thermal, and gelling properties of wheat starch. Food Chemistry 112(4): 919–923.

[42] Avérous L, Fringant C, Moro L (2001) Starch-based biodegradable material suitable for thermoforming packaging. Starch 53: 368–71.

[43] Jansson A, Thuvander F (2004) Influence of thickness on the mechanical properties for starch films. Carbohydrate Polymers 56: 499–503.

[44] Siracusa V (2012) Food packaging permeability behaviour: A report. International Journal of Polymer Science 2012: 1–11.

[45] Immel S, Lichtenthaler FW (2000) The Hydrophobic Topographies of Amylose and its Blue Iodine Complex. Starch/Stärke 52(1): 1–8.

[46] Curcio M, Puoci F, Iemma F, Parisi OI, Cirillo G, et al (2009) Covalent insertion of antioxidant molecules on chitosan by a free radical grafting procedure. Journal Agricultural Food Chemistry 57: 5933–8. 10.1021/jf900778u 19566085

[47] Gumel AM, Annuar MSM, Heidelberg T (2012) Biosynthesis and characterization of polyhydroxyalkanoates copolymers produced by Pseudomonas putida Bet001 isolated from palm oil mill effluent. Plos One 7(9): e45214. 23028854 10.1371/journal.pone.0045214 PMC3447943

[48] Schlemmer D, Oliveira ER, Sales MJA. Polystyrene/thermoplastic starch blends with different plasticizers. Journal of Thermal Analysis and Calorimetric 87(3): 635–638.

[49] Cao G, Sofic E, Prior RL (1997) Antioxidant and prooxidant behavior of flavonoids: structure-activity relationships. Free Radical Biology and Medicine 22: 749–60. 9119242 10.1016/s0891-5849(96)00351-6

[50] Heim K, Tagliaferro AR, Bobilya DJ (2002) Flavonoid antioxidants: chemistry, metabolism and structure-activity relationships. Journal Nutrition Biochemistry 13: 572–84. 10.1016/s0955-2863(02)00208-5 12550068

[51] Xiao X, Shi D, Liu L, Wang J, Xie X, et al (2011) Quercetin suppresses cyclooxygenase-2 expression and angiogenesis through inactivation of P300 signaling. Plos One 6(8): e22934. 21857970 10.1371/journal.pone.0022934 PMC3152552

[52] Munoz-Munoz JL, García-Molina F, Molina-Alarcón M, Tudela J, García-Cánovas F, et al (2008) Kinetic characterization of the enzymatic and chemical oxidation of the catechins in green tea. Journal Agricultural Food Chemistry 56: 9215–24. 10.1021/jf8012162 18788750

[53] Silva JBA, Pereira FV, Druzian JI (2012) Cassava starch-based films plasticized with sucrose and inverted sugar and reinforced with cellulose nanocrystals. Journal of Food Science 77: 14–19. 10.1111/j.1750-3841.2012.02710.x 22582979

[54] Haugaard VK (2001) Potential food applications of biobased materials. An EU-concerted action project. Starch/Stärke 5: 189–200.

[55] Singh A, Sharma PK, Malviya R (2011) Eco friendly pharmaceutical packaging material. World Applied Sciences Journal 14 (11): 1703–1716.

[56] Wei B, Xu X, Jin Z, Tian Y (2014) Surface Chemical Compositions and Dispersity of Starch Nanocrystals Formed by Sulfuric and Hydrochloric Acid Hydrolysis. Plos One 9(2): e86024. 24586246 10.1371/journal.pone.0086024 PMC3937268

